# Significant differences in intestinal fungal community of hooded cranes along the wintering periods

**DOI:** 10.3389/fmicb.2022.991998

**Published:** 2022-09-07

**Authors:** Yuannuo Wu, Zihan Li, Jingru Zhao, Zhong Chen, Xingjia Xiang

**Affiliations:** ^1^School of Resources and Environmental Engineering, Anhui University, Hefei, China; ^2^International Collaborative Research Center for Huangshan Biodiversity and Tibetan Macaque Behavioral Ecology, Hefei, China; ^3^Anhui Province Key Laboratory of Wetland Ecosystem Protection and Restoration, Hefei, China

**Keywords:** hooded crane, gut fungal community, wintering stage, high-throughput sequencing, pathogen

## Abstract

The intestinal microbiota play vital roles for health of wild birds in many ways. Migratory birds with unique life history might increase the risk of pathogenic transmission across the regions. However, few studies have clarified the fungal community structure and inferred the potential pathogens in guts of migratory birds. The high-throughput sequencing method was applied to analyze the fungal community structure and detect the potential fungal pathogens in guts of hooded cranes among different wintering stages. Significant differences were found in gut fungal community composition of hooded cranes among three wintering stages, with the lowest fungal diversity in the late wintering stage. In the late stage, hooded cranes harbored higher relative abundance of plant saprotroph, contributing to food digestion for hosts. Hooded cranes were associated with the lowest diversity and relative abundance of animal pathogens in the late wintering stage. There was an increasing trend of deterministic process for gut fungal community assembly, suggesting that hosts interaction with their fungal communities changed by enhanced gut selection/filtering along wintering periods. Hooded crane was associated with the strongest gut selection/filtering to obtain defined gut fungal community with retaining probiotics (i.e., plant saprotroph) and exclusion of certain pathogens in the late wintering stage. Overall, these results demonstrated that hooded cranes might regulate their gut microbiota to enhance digestion and decrease gut pathogens in preparation for long-term migration.

## Introduction

The guts of vertebrates contain multiple and complex microbial communities (Walter et al., 2011). Intestinal microbiota have numerous functions for their hosts (Li et al., 2016), such as maintaining nutrition (Duca et al., 2021), regulating metabolism and immunity (Sonnenburg and Bäckhed, 2016; Behary et al., 2021). In addition, the gut microbiota are highly dynamic, and plenty factors can significantly affect the intestinal microbial community (Chi et al., 2021), including diet (David et al., 2014), season (Maurice et al., 2015), sex (Org et al., 2016), life style (Vujkovic-Cvijin et al., 2020) and environment (Rothschild et al., 2018).

Avian gut microbiota differ from the majority of vertebrates, as they have extremely complex and unique diet, physiological characteristics and developmental strategy (Song et al., 2020). Correlative network analyses have confirmed the existence of a diverse and complex microbial network in the gut of birds, and the members within microbial network harbored certain interaction (Trosvik and de Muinck, 2015; Paul et al., 2021). Previous study has shown that bird host species are the strongest factor determining their gut microbial community composition (Waite and Taylor, 2014). The diet also has a primary effect on avian gut microbial community (Pan and Yu, 2014). Migratory birds inhabit different environments during their annual cycling, and their diets vary greatly throughout the year, with fluctuation in their gut microbial composition (Grond et al., 2019). The diet can also influence microbial diversity of migratory birds by mediating community assembly process (Youngblut et al., 2019).

As migratory birds would travel long distances and use different habitats before reaching the wintering site, they might contact various pathogens and might be regarded as important vectors of infectious pathogens (Ryu et al., 2014). These pathogens in migratory birds could be transmitted among wintering birds when they forage together among wintering periods (Bodawatta et al., 2022). Studies have shown that migratory birds would pose certain risks to animals and even human health by the spread of disease (Sturm-Ramirez et al., 2005; Zhao et al., 2018). Therefore, understanding the potential pathogens in guts of migratory birds might be helpful to clarify the potential pathogenic transmission cross species.

The Shengjin Lake is an internationally important wetland, offering perfect habitats for migratory birds during wintering periods (Xiang et al., 2019). Hooded crane is one of large long-distance wading wild birds, belong to Threatened Species in the IUCN (International Union for Conservation of Nature and Natural Resources) Red List and the first-class national protected wild animal in China (Zheng et al., 2015). Current studies have focused on the bacterial community structure and their roles in health or disease of hooded cranes (Xiang et al., 2019; Fu et al., 2020). However, the ecological roles or potential pathogens of gut fungal community in hooded cranes have not been well estimated.

The study of gut microbiota was helpful to better understand avian wintering ecology and their environmental adaptation. In this study, the high-throughput sequencing method was used to analyze the gut fungal community structure and infer the potential pathogens in guts of hooded cranes among different wintering stages. The aims of this study were (1) to clarify the effect of wintering periods on gut fungal community structure, (2) to evaluate the potential functions of fungal community for hooded cranes, and (3) to identify potential pathogens in guts of hooded cranes in different wintering stages.

## Materials and methods

### Sample collection and DNA extraction

The study site was located in the Shengjin lake, an internationally important wetland. The Shengjin lake served as an indispensable wintering area for migratory birds (Fox et al., 2011). According to the natural climatic characteristics of the lake, the wintering period was divided into three stages, named the early, middle and late stages (Wei et al., 2020). Fecal samples were collected from three wintering stages: the early stage from November 1st to November 2nd, 2018; the middle stage from December 28th to December 29th, 2018; the late stage from February 19th to February 20th, 2019. Before sampling, the telescope was used to search the flocks of hooded crane. Fresh fecal samples were collected immediately after foraging. DNA extractions of fecal samples were performed by using the Qiagen DNA Stool Mini Kit.

### Bird species determination

Primers BIRDF1/BIRDR1 were used to amplify the COL gene to identify bird species (Hebert et al., 2004). PCR reaction was carried out in 50 μl reaction mixtures, with the parameters as follows: 95°C for 5 min, a total of 35 cycles, 95°C for 30 s, 55°C for 45 s, and 72°C for 90 s, with a final extension period at 72°C for 10 min. The PCR products was sequenced and blasted (>99% identity) in National Center for Biotechnology Information (NCBI) to identify bird species. Only hooded crane samples were retained for high-throughput sequencing.

### Bioinformatics

PCR reaction was conducted using primer ITS1/ITS2 for sequencing to get the fungal data. Raw data were analyzed by QIIME software (QIIME 1.9; Caporaso et al., 2010). High-quality sequences (average mass fraction more than 30 and length > 250 bp) were clustered into Operational Taxonomic Units (OTU) by the UCLUST (Edgar, 2010). OTUs of chimera and monomer were deleted. The most abundant sequence within each OTU was selected as the representative sequence identified by the ribosomal database project Classifier, and aligned by PyNAST (Caporaso et al., 2010). Randomly selecting subsets of 43,000 sequences per sample were used to compare fungal community compositions and diversity for all samples.

### Statistical analysis

The differences of fungal community compositions were shown by non-metric multidimensional scaling (NMDS). Biomarkers of intestinal fungi in each stage were identified by linear discriminant analysis (LDA) effect size (Segata et al., 2011). The fungal functional guilds (i.e., functional group) were assigned using the FUNGuild pipeline (Nguyen et al., 2016). The abundance-based β-null model was used to distinguish the relative importance of deterministic and stochastic processes by the value of null deviation (NDV, Luan et al., 2020). Co-occurrence network analysis was used to clarify the interaction of intestinal fungi in guts of hooded cranes among three wintering stages. The detail information of Materials and Methods can be found in Supporting Information.

## Results

### Gut fungal alpha-diversity

**Figure 1:**
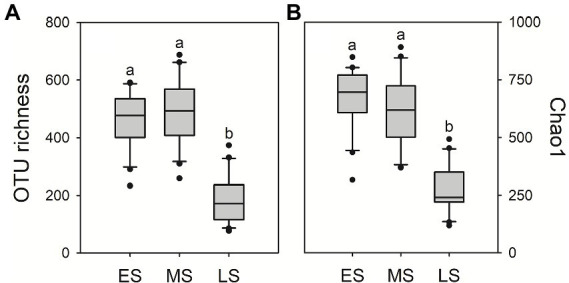
Intestinal fungal alpha-diversity in the three stages of hooded crane. Gut fungal OTU richness **(A)** and Chao1 index **(B)**. The bottom and top of the box denote the first and third quartiles; the band inside the box denotes median. Letters represent significant differences from the One-way ANOVA test (*P* < 0.05). ES: early stage; MS: middle stage; LS: late stage. OTU: operational taxonomic units. ANOVA: Analysis of Variance.

A total of 3,754,368 quality-filtered fungal sequences and a total of 3,469 fungal OTUs were detected in all samples, ranging from 43,786 to 72,614 sequences and 73 to 688 OTUs per sample, respectively. There were 574 OTUs (16.5%) sharing in guts of hooded cranes across three wintering stages (Supplementary Figure S1). The unique gut fungal OTUs were 607 (17.5%), 1,093 (31.5%) and 191 (5.5%) for the early, middle and late stages, respectively. The result of One-way ANOVA showed that gut fungal alpha-diversity (i.e., OTU richness and Chao1 index) was the lowest in the late stage compared to early and middle stages (Figure 1).

### Gut fungal community structure

**Figure 2:**
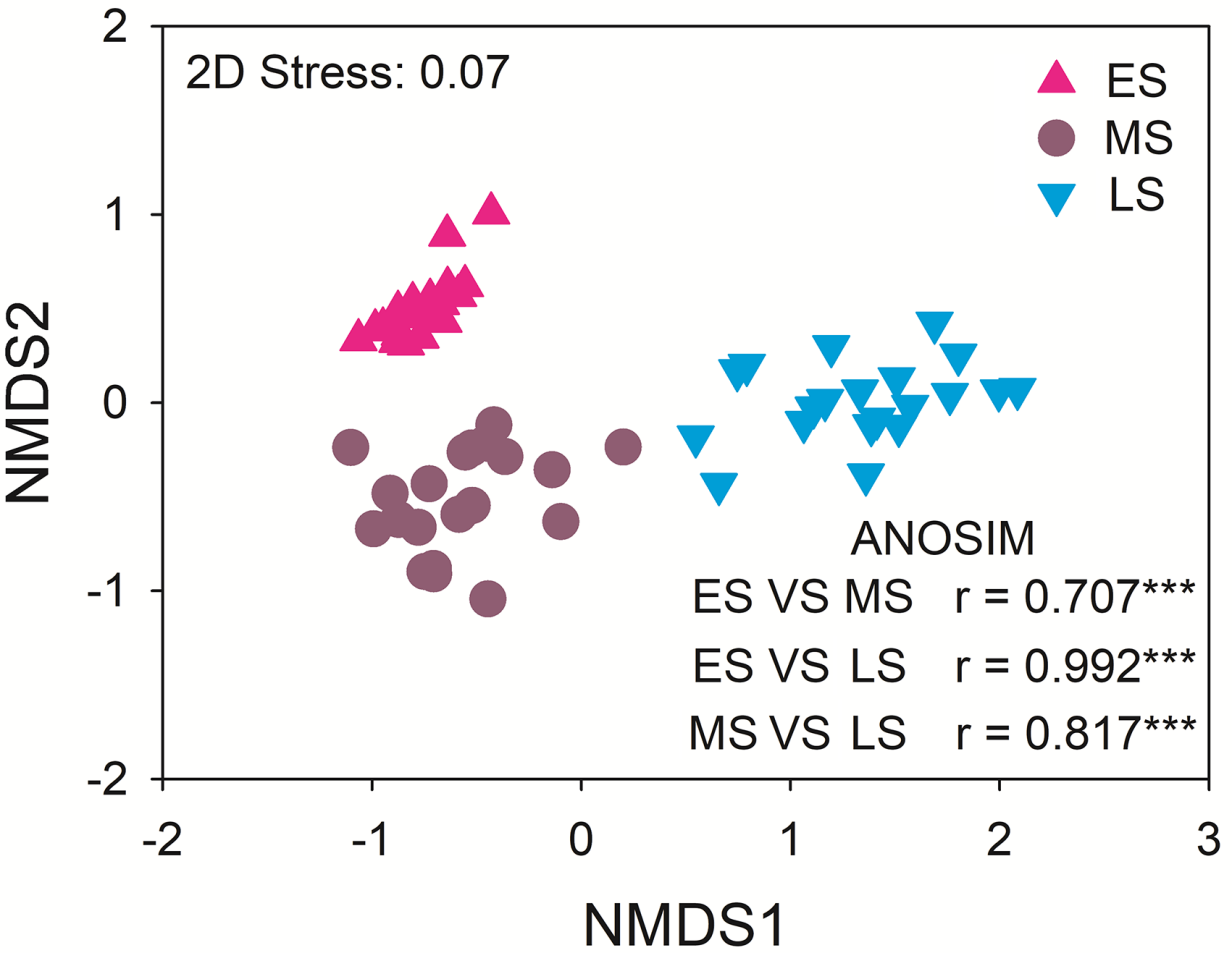
The intestinal fungal community composition among the three stages. ES: early stage; MS: middle stage; LS: late stage. ANOSIM: analysis of similarity. ***P < 0.001.

The dominant gut fungal phyla were Ascomycota (80.13%), Basidiomycota (11.71%), Zygomycota (5.72%) and Rozellomycota (1.10%). The late stage had the highest relative abundance of Ascomycota. The middle stage had higher relative abundance of Basidiomycota and Rozellomycota relative to early and late stages. The relative abundance of Zygomycota was the highest in the early stage (Supplementary Figure S2). There were significant differences in the gut fungal community composition of hooded cranes among three wintering stages (*p* < 0.001 in all cases; Figure 2). The abundance-based β-null model was used to distinguish the relative importance of deterministic and stochastic processes (Supplementary Figure S3). The result showed an increasing trend of null deviation value (NDV) with the greater influence of deterministic process along wintering periods.

**Figure 3:**
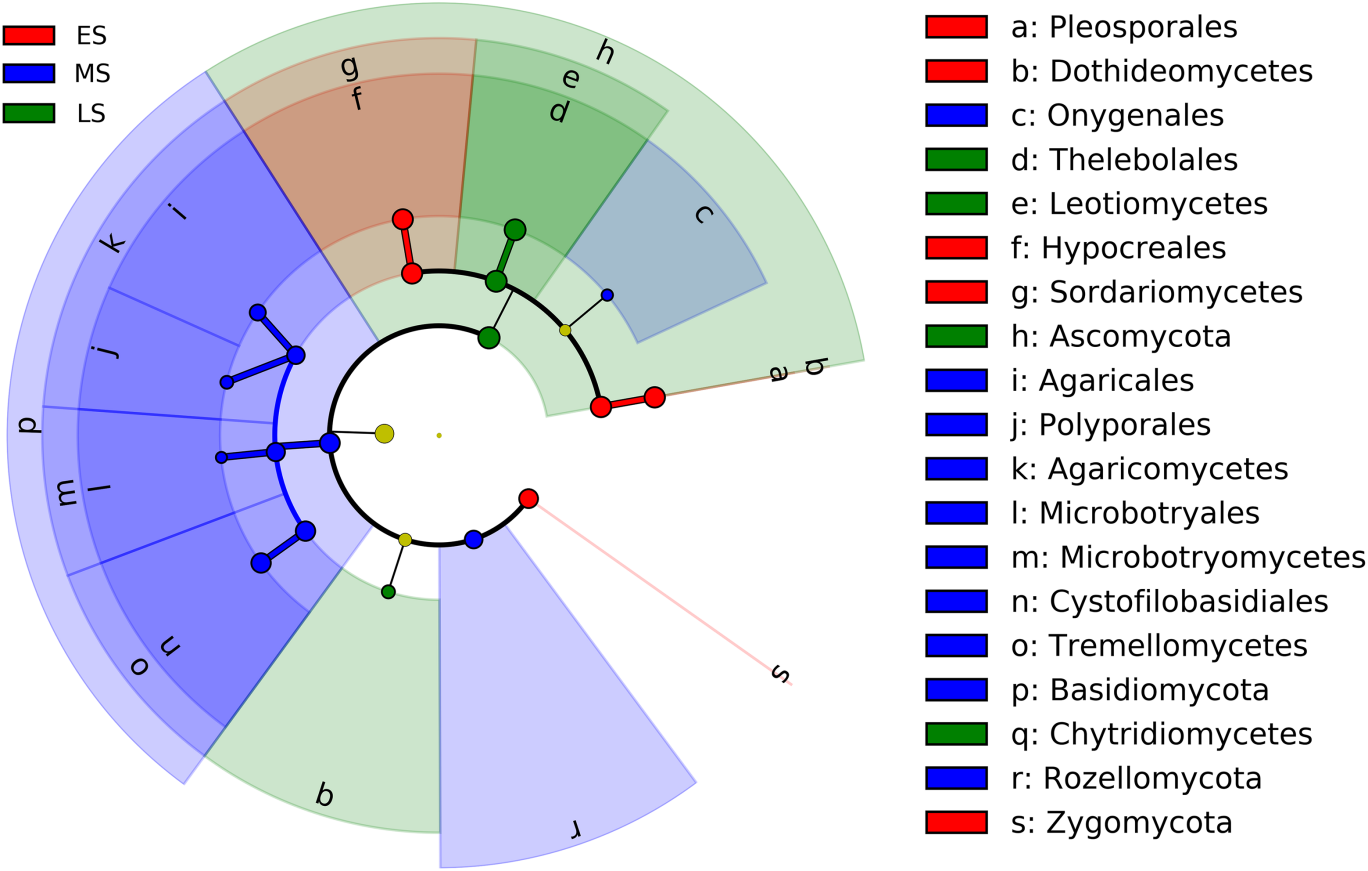
LEfSe analysis showing intestinal fungal biomarkers associated with each stage (the effect size >2 and the alpha value was <0.05). ES, early stage; MS, middle stage; LS, late stage.

The result of LEfSe showed that fungi in one phylum (i.e., Zygomycota), 2 classes (i.e., Dothideomycetes and Sordariomycetes), and 2 orders (i.e., Pleosporales and Hypocreales) were enriched in the early stage (Figure 3; Supplementary Figure S4). Fungi from two phyla (i.e., Basidiomycota and Rozellomycota), three classes (i.e., Agaricomycetes, Microbotryomycetes and Tremellomycetes) and five orders (i.e., Onygenales, Agaricales, Polyporales, Microbotryales and Cystofilobasidiales) were more abundant in the middle stage (Figure 3; Supplementary Figure S4). One phylum (i.e., Ascomycota), 2 classes (i.e., Leotimycetes and Chytridiomycetes), and 1 order (i.e., Theleboales) were enriched in the late stage (Figure 3; Supplementary Figure S4).

There were 12 (e.g., *Phoma*, *Gibberella*, *Mortierella*, etc), 9 (i.e., *Acremonium*, *Cystofilobasidium*, *Rhodotorula*, etc) and 7 (i.e., *Schizothecium*, *Didymella*, *Botrytis*, etc) indicator genera in the early, middle and late stages, respectively (Table 1), and there were 14 (e.g., *Phoma calidophila*, *Gibberella fujikuroi*, *Mortierella camargensis*, etc), 13 (i.e., *Cystofilobasidium infirmominiatum*, *Acremonium dichromosporum*, *Acremonium nepalense*, etc) and 10 (i.e., *Thelebolales* sp., *Schizothecium carpinicola*, *Didymella exigua*, etc) indicator species in the early, middle and late stages, respectively (Supplementary Table S1). Simper analysis indicated that *Phoma calidophila* (21%) and *Gibberella fujikuroi* (16%) contributed largely to the differences of the fungal community composition between the early and middle stages. The *Thelebolales* sp. (37%) and *Phoma calidophila* (18%) primarily triggered the difference of fungal community composition between the early and middle stages. The *Thelebolales* sp. (41%) and *Cystofilobasidium infirmominiatum* (9.1%) were the main taxa explained the difference of fungal community composition between middle and late stages (Supplementary Table S2).

**Table 1:** Indicator analysis was conducted to show indicator genera with relative abundance >0.1% of each stage.

	Indicator value	*P*	Taxonomy	Relative abundance (%)
ES	0.777	0.001	g__*Phoma*	14.29
	0.929	0.001	g__*Gibberella*	7.616
	0.541	0.004	g__*Mortierella*	5.417
	0.519	0.023	g__*Nomuraea*	0.374
	0.706	0.001	g__*Westerdykella*	0.286
	0.722	0.001	g__*Humicola*	0.256
	0.590	0.044	g__*Pyrenochaetopsis*	0.230
	0.746	0.001	g__*Sporobolomyces*	0.171
	0.597	0.001	g__*Talaromyces*	0.171
	0.521	0.016	g__*Aspergillus*	0.118
	0.888	0.001	g__*Clonostachys*	0.118
	0.774	0.001	g__*Myrmecridium*	0.113
MS	0.594	0.001	g__*Acremonium*	10.17
	0.740	0.001	g__*Cystofilobasidium*	6.386
	0.691	0.005	g__*Rhodotorula*	1.857
	0.577	0.001	g__*Ascochyta*	1.106
	0.200	0.040	g__*Sclerotium*	0.207
	0.966	0.001	g__*Leucosporidiella*	0.205
	0.559	0.009	g__*Davidiella*	0.184
	0.796	0.001	g__*Mastigobasidium*	0.146
	0.867	0.001	g__*Zopfiella*	0.104
LS	0.884	0.001	g__*Schizothecium*	2.172
	0.678	0.016	g__*Didymella*	0.584
	0.891	0.001	g__*Botrytis*	0.526
	0.762	0.013	g__*Alternaria*	0.482
	0.617	0.001	g__*Cryptococcus*	0.395
	0.731	0.001	g__*Leucosporidium*	0.269
	0.758	0.003	g__*Preussia*	0.124

ES, early stage; MS, middle stage; LS, late stage.

### Gut potential saprotroph, pathogens and endosymbionts

**Figure 4:**
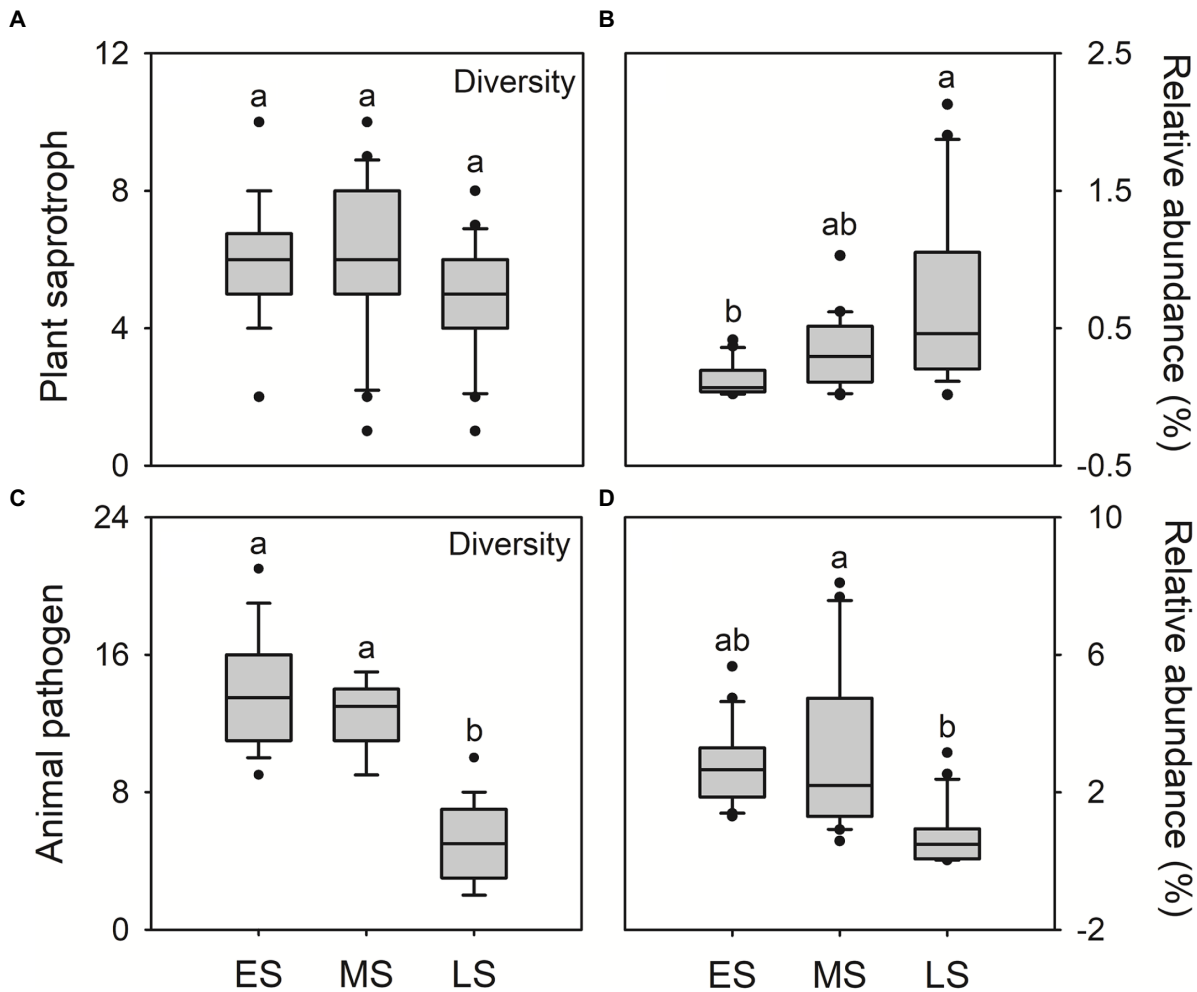
The plant saprotrophic diversity (i.e., saprotrophic OTU; **A**); the relative abundance of plant saprotroph **(B)**; animal pathogenic diversity (i.e., pathogenic OTU; **C**); the relative abundance of animal pathogen **(D)**. Letters represent significant differences from the Kruskal-Wallis test (*p* < 0.05). ES, early stage; MS, middle stage; LS, late stage. OTU, operational taxonomic units.

The FUNGuild analysis was used to infer the plant saprotroph, animal pathogens and endosymbionts in guts of hooded cranes. A total of 12 plant saprotrophic OTUs, 28 animal pathogenic OTUs and 10 animal endosymbiotic OTUs were detected across all samples, ranging from 1 to 10, 2 to 21 and 0 to 8 OTUs per sample, respectively (Supplementary Figure S5). There was litter difference in gut saprotrophic and endosymbiotic community composition among three wintering stages. The gut pathogenic community composition in the late stage was significantly different from that in early and middle stages (Supplementary Figure S6). There was little difference in saprotrophic diversity among three wintering stages (Figure 4A). In the late stage, hooded cranes had the highest relative abundance of plant saprotroph (Figure 4B), the lowest diversity and relative abundance of animal pathogens and endosymbionts relative to other stages (Figures 4C,D; Supplementary Figure S7).

### Gut fungal network analysis

Co-occurrence network analysis was used to understand the interaction within intestinal fungal taxa of hooded cranes among three wintering stages. Generally, the majority of links in the intestinal network were related to Ascomycota (Supplementary Figure S8). The late stage had the highest clustering coefficient and average degree, the lowest average path length and modularity relative to early and middle stages (Supplementary Table S3). The network stability was assessed by natural connectivity, with the highest network stability in the late stage compared to early and middle stages (Supplementary Figure S9). The different roles of network nodes were identified by Zi-Pi plots (Supplementary Figure S10). There were 33, 35 and 20 keystone fungal OTUs in the early, middle and late stages, respectively (Supplementary Table S4).

## Discussion

Empirical studies have demonstrated that seasonal variation triggered dramatic shifts in animal gut microbial community (Maurice et al., 2015; Ren et al., 2017), which was consistent with this study. In addition, we found an increasing tread of deterministic process in gut fungal community assembly along wintering periods. Microbial community structure could be shaped by stochastic or deterministic processes (Yuan et al., 2019). The increasing relative importance of deterministic process might strengthen the role of environmental selection (Stegen et al., 2012). Gut filtering was an important selection factor to influence the animal microbial community structure (Zhou and Ning, 2017; Yang et al., 2021). The strong gut filtering would select specific microbial taxa and exclude certain taxa in the intestinal ecosystem, triggering the decrease of microbial diversity (Costello et al., 2012; Yan et al., 2016). Thus, the increasing effect of gut filtering might lead to the changes of fungal community composition and the decrease of fungal diversity of hooded cranes along wintering periods (Figures 1, 2).

The co-occurrence network showed the most complexity with the highest stability, average degree and the lowest average path length in late wintering stage (Supplementary Figure S9; Supplementary Table S3). The network with more stability might be associated with stronger resistance to environmental fluctuations and support greater multifunctionality (Deng et al., 2016; Yang et al., 2021). The higher average degree and lower average path length contributed to better organized pathways for metabolites and information exchange among gut fungal taxa (Zhou et al., 2010). The Ascomycota exhibited the major links of co-occurrence network and acted as keystone taxa in guts of hooded cranes (Supplementary Figure S8; Supplementary Table S4). We also found that the highest relative abundance of Ascomycota in the late wintering stage (Supplementary Figure S2A). Ascomycota were the main cellulolytic fungi (Zheng et al., 2020), and they could secrete massive cellulase and hemicellulase to decompose complex polysaccharides (Lichius et al., 2015; Linton, 2020; Sun et al., 2022) and improve the nutrient utilization rate for their hosts (Zhang et al., 2019).

In this study, the relative abundance of plant saprotroph was higher in the late wintering stage compared to early and middle stages (Figure 4B). Previous studies have shown that hooded cranes mainly forage *Vallisneria spinulosa* and *Potamogeton malaianus* at Shengjin lake in wintering periods (Wei et al., 2020; Xiang et al., 2021). Therefore, the higher relative abundance of plant saprotroph might be related to the rapid digestion of food for hooded cranes. Moreover, the *Alternaria* and *Preussia* were detected as indicator genera in the late stage (Table 1). The two enriched genera have been demonstrated to facilitate the digestion of food (Sugino, 2007; Sun et al., 2011; Liang et al., 2015). Overall, these results demonstrated that, in late wintering stage, hooded cranes might depend on their gut microbiota to enhance digestion and acquire more nutrients from food in preparation for long-term migration.

We also detected the potential pathogens in guts of hooded cranes. Hooded cranes might suffer from various pathogenic invasion to cause inflammation during wintering periods (Xiang et al., 2019). A total of 28 potentially pathogenic OTUs has been detected in the gut of hooded cranes (Supplementary Figure S4B), with higher diversity and relative abundance in early and middle stages than in the late stage (Figures 4C,D; Supplementary Figure S7). Diseases could interrupt appropriate intestinal function to reduce the co-nutrition interaction within gut fungal taxa, which might be a reason resulting in the intestinal network with lower stability in early and middle stages (Zhu et al., 2016; Supplementary Figure S9). In addition, the *Cordyceps bassiana* was detected as keystone species in the late stage (Supplementary Table S4). Related studies have shown that the *Cordyceps bassiana* showed anti-inflammatory potential and improved immunity for hosts (Wu et al., 2011; Yang et al., 2015). Thus, these results suggested that intestinal certain fungal taxa might increase hosts’ immunity to resist pathogens for hooded cranes in the late wintering stage.

In this study, hooded cranes suffered invasion from a variety of pathogens under harsh living environment (Supplementary Table S5). More attention should be paid to protect hooded cranes as they are endangered species. These pathogens in hooded cranes might cause a series of diseases not only for birds, but also other animals, even humans (Supplementary Table S5). Previous study has shown that wintering hooded cranes often foraged with poultry at Shengjin lake (Xiang et al., 2019). Therefore, the overlap of foraging niches could trigger pathogenic transmission from hooded cranes to poultry. Notably, poultry could in turn act as vectors to spread pathogens to local residents. Thus, more studies should be paid to clarify the underlying pathway of pathogenic transmission from wild migratory birds to poultry and human beings.

## Conclusion

In summary, the increasing gut filtering might trigger the significant differences in fungal community composition of hooded cranes along wintering periods, with the strongest gut selection/filtering to retain probiotics (i.e., Ascomycota and plant saprotroph) and exclude certain pathogens in the late wintering stage. The results suggested that hooded cranes might regulate their gut fungal community to enhance digestion and immunity in preparation for long-term migration. This work contributed to a more complete picture of the ecological function of the gut microbiota in migratory birds. However, there were certain limitations in this study. Previous studies have demonstrated that sex and age were important factors to shape animal gut microbial communities (O’Toole and Jeffery, 2015; Org et al., 2016). We have not yet studied the effects of sex and age of hooded cranes on gut fungal communities. These limitations should be clarified in future studies.

## Data availability statement

The datasets presented in this study can be found in online repositories. The names of the repository/repositories and accession number(s) can be found at: https://www.ncbi.nlm.nih.gov/, SRP383975.

## Author contributions

YW, ZC, and XX designed the experiments. YW, ZL, and JZ completed the field sampling. YW and XX performed the data analysis and prepared the figures. YW wrote the manuscript. XX and ZC contributed to the revision of manuscript. All authors contributed to the article and approved the submitted version.

## Funding

This work was supported by the National Natural Science Foundation of China (31801989), and Anhui University Scientific Research Foundation (S020118002/038).

## Conflict of interest

The authors declare that the research was conducted in the absence of any commercial or financial relationships that could be construed as a potential conflict of interest.

## Publisher’s note

All claims expressed in this article are solely those of the authors and do not necessarily represent those of their affiliated organizations, or those of the publisher, the editors and the reviewers. Any product that may be evaluated in this article, or claim that may be made by its manufacturer, is not guaranteed or endorsed by the publisher.
