# Supplementary material for: Significant differences in intestinal fungal community of hooded cranes along the wintering periods
Source: Front Microbiol. 2022 Sep 7;13:991998. doi: 10.3389/fmicb.2022.991998 (PMC9491237; doi:10.3389/fmicb.2022.991998)
Supplement: Supplementary file 1 [file Data_Sheet_1.docx]

**Supporting Information**

**Supporting Materials and Methods**

**Site selection and sample collection**

The study site was selected in the area of Shengjin lake, an internationally important wetland, connected with the Yangtze River, serving as an indispensable wintering area for migratory birds (Fox et al., 2011). Fecal samples were collected from 2018 to 2019 at the Shegan region, Shengjin lake. According to the natural climatic characteristics of the lake, the wintering period was divided into three stages, named the early, middle and late stages (Wei et al., 2020). The samples were carried out in three stages: the early stage from November 1st to November 2nd, 2018; the middle stage from December 28th to December 29th, 2018; the late stage from February 19th to February 20th, 2019. Before sampling, we first used the telescope to search the flocks of hooded crane. Fresh fecal samples were collected immediately after foraging, the interval distance for fecal samples was > 5m to avoid individual repetition. Fecal samples were quickly transported to the lab and stored at -20°C.

**DNA extraction**

DNA extractions of fecal samples were used the Qiagen DNA Stool Mini Kit according to the DNA isolation protocol. The extracted DNA was dissolved in 60 μl of elution buffer, quantified by NanoDrop ND-1000 (Thermo Scientific, USA), and stored at -20 °C.

**PCR and amplicon library preparation**

PCR reaction was carrying out using primer ITS1/ITS2 in 50 μl mixtures with parameters as follows: 35 cycles of denaturation at 95 °C for 45 s, annealing at 56 °C for 45 s, and extension at 72 °C for 45 s; with a final extension at 72 °C for 10 min. The PCR products were purified for sequencing.

**Statistical analysis**

One-way ANOVA was conducted to evaluate the differences of fungal alpha-diversity (normal distribution, Kolmogorov-Smirnov test) during the three stages for hooded crane. The differences of fungal community composition in three stages were shown by non-metric multidimensional scaling (NMDS) and similarity analysis (ANOSIM; permutations = 999) using vegan package in R software. Biomarkers of intestinal fungi in each stage were identified by linear discriminant analysis (LDA) effect size (Segata et al., 2011). Indicator analysis was conducted to show the enriched genera in each stage, and simper analysis was used to assess the contribution of specific species to the difference between wintering stages. The fungal functional guilds (i.e., functional group) were assigned using the FUNGuild pipeline, and only these guilds with high confidence ranking (i.e., highly probable and probable) were selected for further analysis (Nguyen et al., 2016). The Kruskal-Wallis test was performed to compare the relative abundance of animal pathogen and pathogenic diversity (non-normal distribution) during the three stages.

The abundance-based β-null model was used to distinguish the relative importance of deterministic and stochastic processes by the value of null deviation (Luan et al., 2020). The deterministic process based on ecological niches involves selection imposed under biotic and abiotic conditions (Zhou and Ning, 2017). In contrast, stochastic processes encompassed random births or deaths, ecological drift and probabilistic dispersal (Ning et al., 2019). Deterministic and stochastic process would act concurrently when constructing ecological community (Zhou et al., 2014). The shifts of selection intensity and diffusion rate could affect the relative importance of deterministic and stochastic processes in ecosystem (Tripathi et al., 2018).

Co-occurrence network analysis was used to understand the interaction in intestinal fungi of hooded cranes among three wintering stages, the topological characteristics of fungal network were calculated to decode the complex symbiotic model among fungus. The network stability was estimated by removing nodes from the static network and evaluating the speed of robustness degradation (Fan et al., 2018). The network nodes were divided into four categories based on within module connectivity and among module connectivity according to their network roles, including module hubs (Zi-score > 2.5; Pi-score < 0.62), network hubs (Zi-score > 2.5; Pi-score > 0.62), connectors (Zi-score < 2.5; Pi-score > 0.62) and peripherals (Zi-score < 2.5; Pi-score < 0.62) in network structure (Poudel et al., 2016). Nodes belonging to module hubs, network hubs and connectors were considered as keystone species in intestinal microbial community (Li et al., 2018).

**Supporting reference:**

Fan K, Weisenhorn P, Gilbert JA, Shi Y, Bai Y, Chu HY. Soil pH correlates with the co-occurrence and assemblage process of diazotrophic communities in rhizosphere and bulk soils of wheat fields. Soil Biology and Biochemistry. 2018; 121: 185-192. doi: 10.1016/j.soilbio.2018.03.017

Fox AD, Cao L, Zhang Y, Barter M, Zhao MJ, Meng FJ, Wang SL. Declines in the tuber-feeding waterbird guild at Shengjin Lake National Nature Reserve, China-a barometer of submerged macrophyte collapse. Aquatic Conservation: Marine and Freshwater Ecosystems. 2011; 21: 82-91. doi: 10.1002/aqc.1154

Li H, Li TT, Li XZ, Wang GH, Lin Q, Qu JP. Gut microbiota in Tibetan herdsmen reflects the degree of urbanization. Frontiers in Microbiology. 2018; 9: 1745. doi: 10.3389/fmicb.2018.01745

Luan L, Liang C, Chen LJ, Wang HT, Xu QS, Jiang YJ, Sun B. Coupling bacterial community assembly to microbial metabolism across soil profiles. mSystems. 2020; 5: e00298-20. doi: 10.1128/mSystems.00298-20

Nguyen NH, Song Z, Bates ST, Branco S, Tedersoo L, Menke J, Schilling JS, Kennedy PG. FUNGuild: an open annotation tool for parsing fungal community datasets by ecological guild. Fungal Ecology. 2016; 20: 241-248. doi: 10.1016/j.funeco.2015.06.006

Ning DL, Deng Y, Tiedje JM, Zhou JZ. A general framework for quantitatively assessing ecological stochasticity. Proceedings of the National Academy of Sciences. 2019; 116: 16892-16898. doi: 10.1073/pnas.1904623116

Poudel R, Jumpponen A, Schlatter DC, Paulitz TC, Gardener BBM, Kinkel LL, Garrett KA. Microbiome networks: a systems framework for identifying candidate microbial assemblages for disease management. Phytopathology. 2016; 106: 1083-1096. doi: 10.1094/PHYTO-02-16-0058-FI

Segata N, Izard J, Waldron L, Gevers D, Miropolsky L, Garrett WS, Huttenhower C. Metagenomic biomarker discovery and explanation. Genome Biology. 2011; 12: 1-18. doi: 10.1186/gb-2011-12-6-r60

Tripathi BM, Stegen JC, Kim M, Dong K, Adams JM, Lee YK. Soil pH mediates the balance between stochastic and deterministic assembly of bacteria. The ISME Journal. 2018; 12: 1072-1083. doi: 10.1038/s41396-018-0082-4

Wei ZH, Zheng M, Zhou LZ, Xu WB. Flexible foraging response of wintering hooded cranes (*Grus monacha*) to food availability in the lakes of the Yangtze River floodplain, China. Animals. 2020; 10: 568. doi: 10.3390/ani10040568

Zhou JZ, Deng Y, Zhang P, Arkin AP. Stochasticity, succession, and environmental perturbations in a fluidic ecosystem. Proceedings of the National Academy of Sciences. 2014; 111: E836-E845. doi: 10.1073/pnas.1324044111

Zhou JZ, Ning DL. Stochastic community assembly: does it matter in microbial ecology? Microbiology and Molecular Biology Reviews. 2017; 81: e00002-17. doi: 10.1128/MMBR.00002-17

**Table S1:** Indicator analysis was conducted to show indicator species with relative abundance > 0.1% of each stage. ES: early stage; MS: middle stage; LS: late stage.

|  | Indicator value | *P* | Taxonomy | Relative abundance  (%) |
| --- | --- | --- | --- | --- |
| ES | 0.777 | 0.001 | s*__Phoma calidophila* | 14.28 |
|  | 0.938 | 0.001 | s*__Gibberella fujikuroi* | 7.526 |
|  | 0.545 | 0.008 | s*__Mortierella camargensis* | 5.225 |
|  | 0.519 | 0.028 | s*__Nomuraea rileyi* | 0.374 |
|  | 0.496 | 0.005 | s*__Pleosporales* sp. | 0.347 |
|  | 0.516 | 0.035 | s*__Penicillium abidjanum* | 0.340 |
|  | 0.706 | 0.001 | s__*Westerdykella dispersa* | 0.282 |
|  | 0.781 | 0.001 | s__*Humicola nigrescens* | 0.233 |
|  | 0.487 | 0.041 | s__*Xenopolyscytalum* sp. | 0.179 |
|  | 0.627 | 0.001 | s__*Talaromyces marneffei* | 0.159 |
|  | 0.783 | 0.001 | s__*Sporobolomyces ruberrimus* | 0.159 |
|  | 0.866 | 0.001 | s__*Penicillium levitum* | 0.159 |
|  | 0.891 | 0.001 | *s__Clonostachys rosea f. catenulata* | 0.117 |
|  | 0.774 | 0.001 | s__*Myrmecridium* sp. | 0.113 |
| MS | 0.740 | 0.001 | s*__Cystofilobasidium infirmominiatum* | 6.386 |
|  | 0.672 | 0.001 | s*__Acremonium dichromosporum* | 5.262 |
|  | 0.517 | 0.003 | s__*Acremonium nepalense* | 4.820 |
|  | 0.693 | 0.001 | s*__Rhodotorula vanillica* | 1.848 |
|  | 0.577 | 0.001 | s*__Ascochyta maackiae* | 1.106 |
|  | 0.955 | 0.001 | s*__Rozellomycota* sp. | 1.103 |
|  | 0.398 | 0.038 | s__*Ceratobasidiaceae* sp. | 0.761 |
|  | 0.598 | 0.007 | s__*Penicillium oxalicum* | 0.423 |
|  | 0.870 | 0.001 | s__*Mortierellales* sp. | 0.254 |
|  | 0.566 | 0.049 | s__*Stagonospora perfecta* | 0.224 |
|  | 0.966 | 0.001 | s__*Leucosporidiella fragaria* | 0.205 |
|  | 0.559 | 0.005 | s__*Davidiella tassiana* | 0.184 |
|  | 0.796 | 0.001 | s__*Mastigobasidium intermedium* | 0.146 |
| LS | 0.864 | 0.001 | s*__Thelebolales* sp. | 27.86 |
|  | 0.911 | 0.001 | s*__Schizothecium carpinicola* | 2.072 |
|  | 0.678 | 0.017 | s*__Didymella exigua* | 0.584 |
|  | 0.891 | 0.001 | s*__Botrytis caroliniana* | 0.526 |
|  | 0.763 | 0.014 | s*__Alternaria* sp. | 0.481 |
|  | 0.764 | 0.002 | s*__Sporormiaceae* sp. | 0.452 |
|  | 0.710 | 0.001 | s__*Cryptococcus gastricus* | 0.329 |
|  | 0.872 | 0.001 | s__*Ascobolaceae* sp. | 0.323 |
|  | 0.937 | 0.001 | s__*Leucosporidium golubevii* | 0.185 |
|  | 0.728 | 0.007 | s__*Preussia flanaganii* | 0.114 |

**Table S2:** Simper analysis was used to assess the contribution of specific species to dissimilarity of gut fungal community composition between the two wintering stages. ES: early stage; MS: middle stage; LS: late stage.

| Taxonomy | Contribution (%)  ES VS MS |
| --- | --- |
| s__*Phoma calidophila* | 21.10 |
| s__*Gibberella fujikuroi* | 16.29 |
| s__*Cystofilobasidium infirmominiatum* | 9.240 |
| s__*Thelebolales* sp. | 9.010 |
| s__*Acremonium dichromosporum* | 6.790 |
|  | ES VS LS |
| s__*Thelebolales* sp. | 36.69 |
| s__*Phoma calidophila* | 18.19 |
| s__*Gibberella fujikuroi* | 11.64 |
| s__*Mortierella camargensis* | 4.010 |
| s__*Acremonium nepalense* | 3.800 |
|  | MS VS LS |
| s__*Thelebolales* sp. | 40.59 |
| s__*Cystofilobasidium infirmominiatum* | 9.100 |
| s__*Phoma calidophila* | 6.150 |
| s__*Acremonium dichromosporum* | 5.630 |
| s__*Acremonium nepalense* | 4.850 |

**Table S3:** Co-occurrence network topological features statistics. ES: early stage; MS: middle stage; LS: late stage.

| **Stages** | **ES** | **MS** | **LS** |
| --- | --- | --- | --- |
| **Nodes** | 197 | 206 | 191 |
| **Edges** | 984 | 802 | 2658 |
| **Density** | 0.051 | 0.038 | 0.146 |
| **Modularity** | 0.735 | 0.789 | 0.447 |
| **Dgree (Average)** | 9.99 | 7.786 | 27.83 |
| **Clustering coefficient** | 0.65 | 0.68 | 0.73 |
| **Path length (Average)** | 3.885 | 5.066 | 2.762 |
| **Closeness centrality (Average)** | 0.302 | 0.280 | 0.394 |
| **Betweenness centrality (Average)** | 252 | 316 | 159 |
| **Eigenvector centrality (Average)** | 0.178 | 0.167 | 0.320 |

**Table S4:** Classification information of key taxa in intestinal co-occurrence network. ES: early stage; MS: middle stage; LS: late stage.

| Stages | OTU | Zi | Pi | Phylum | Species |
| --- | --- | --- | --- | --- | --- |
| ES | OTU1405 | -1.041 | 0.7500 | Ascomycota | Unclassified |
|  | OTU1650 | -1.105 | 0.8125 | Rozellomycota | *Rozellomycota* sp. |
|  | OTU2647 | -1.041 | 0.7500 | Ascomycota | *Didymella exigua* |
|  | OTU2648 | -1.116 | 0.6875 | Ascomycota | *Phoma calidophila* |
|  | OTU2676 | 0.428 | 0.6327 | Ascomycota | *Acremonium dichromosporum* |
|  | OTU2737 | -0.956 | 0.6875 | Basidiomycota | *Rhodotorula vanillica* |
|  | OTU286 | -1.105 | 0.7500 | Ascomycota | *Didymella exigua* |
|  | OTU290 | 1.155 | 0.7500 | Ascomycota | *Talaromyces marneffei* |
|  | OTU295 | -1.105 | 0.7778 | Basidiomycota | *Cryptococcus gastricus* |
|  | OTU296 | -1.105 | 0.7500 | Basidiomycota | *Sporobolomyces ruberrimus* |
|  | OTU2994 | -1.041 | 0.7500 | Ascomycota | *Phoma calidophila* |
|  | OTU3007 | -1.105 | 0.7500 | Ascomycota | *Schizothecium carpinicola* |
|  | OTU313 | -0.214 | 0.6389 | Ascomycota | *Lecythophora* sp. |
|  | OTU3471 | -0.856 | 0.7778 | Ascomycota | *Thelebolales* sp. |
|  | OTU352 | -0.807 | 0.6389 | Ascomycota | Unclassified |
|  | OTU353 | -0.577 | 0.7500 | Ascomycota | *Penicillium oxalicum* |
|  | OTU377 | -0.212 | 0.6568 | Unclassified | Unclassified |
|  | OTU392 | -0.807 | 0.6389 | Ascomycota | *Pseudeurotium hygrophilum* |
|  | OTU510 | 1.155 | 0.7500 | Ascomycota | Unclassified |
|  | OTU511 | -1.105 | 0.7500 | Unclassified | Unclassified |
|  | OTU532 | -0.866 | 0.7500 | Ascomycota | Unclassified |
|  | OTU535 | -1.324 | 0.7500 | Basidiomycota | *Leucosporidiales* sp. |
|  | OTU669 | -1.324 | 0.7500 | Ascomycota | *Westerdykella dispersa* |
|  | OTU673 | -0.866 | 0.7500 | Basidiomycota | Unclassified |
|  | OTU690 | -1.116 | 0.6389 | Basidiomycota | *Leucosporidiales* sp. |
|  | OTU705 | -1.509 | 0.7551 | Rozellomycota | *Rozellomycota* sp. |
|  | OTU707 | -1.254 | 0.7500 | Basidiomycota | *Leucosporidium* sp. |
|  | OTU709 | -0.856 | 0.7500 | Ascomycota | *Neostagonospora elegiae* |
|  | OTU710 | -1.532 | 0.7500 | Ascomycota | *Gibberella zeae* |
|  | OTU720 | -1.105 | 0.7500 | Basidiomycota | *Sporobolomyces ruberrimus* |
|  | OTU721 | -2.460 | 0.7500 | Ascomycota | *Sarocladium strictum* |
|  | OTU725 | -1.324 | 0.7778 | Ascomycota | *Tetracladium* sp. |
|  | OTU727 | -0.509 | 0.6296 | Ascomycota | Unclassified |
| MS | OTU1580 | 0.000 | 0.7500 | Ascomycota | Unclassified |
|  | OTU1587 | -1.754 | 0.7778 | Ascomycota | *Clonostachys rosea f. catenulata* |
|  | OTU1650 | -1.490 | 0.7500 | Rozellomycota | *Rozellomycota* sp. |
|  | OTU2283 | -1.195 | 0.6875 | Ascomycota | *Pyrenochaetopsis leptospora* |
|  | OTU2663 | -0.871 | 0.7778 | Basidiomycota | *Cystofilobasidium infirmominiatum* |
|  | OTU286 | -0.865 | 0.6875 | Ascomycota | *Didymella exigua* |
|  | OTU287 | -1.484 | 0.6875 | Ascomycota | Unclassified |
|  | OTU288 | -1.195 | 0.7500 | Ascomycota | *Botrytis caroliniana* |
|  | OTU290 | -0.903 | 0.6389 | Ascomycota | *Talaromyces marneffei* |
|  | OTU292 | -0.603 | 0.6700 | Ascomycota | Unclassified |
|  | OTU3023 | -1.774 | 0.7778 | Ascomycota | Unclassified |
|  | OTU307 | -1.023 | 0.7500 | Ascomycota | *Westerdykella dispersa* |
|  | OTU308 | -1.195 | 0.6875 | Ascomycota | *Xenopolyscytalum* sp. |
|  | OTU3479 | 0.000 | 0.7500 | Ascomycota | Unclassified |
|  | OTU350 | -1.302 | 0.7500 | Ascomycota | *Pleosporales* sp. |
|  | OTU3515 | -1.023 | 0.7500 | Ascomycota | *Acremonium dichromosporum* |
|  | OTU358 | -0.871 | 0.7500 | Unclassified | Unclassified |
|  | OTU367 | -2.064 | 0.7778 | Ascomycota | *Cephaliophora* sp. |
|  | OTU372 | -0.998 | 0.6875 | Unclassified | Unclassified |
|  | OTU373 | -1.169 | 0.6296 | Ascomycota | Unclassified |
|  | OTU376 | 0.000 | 0.7500 | Ascomycota | *Pleosporales* sp. |
|  | OTU380 | -0.998 | 0.6875 | Unclassified | Unclassified |
|  | OTU389 | -1.206 | 0.7500 | Ascomycota | *Alternaria* sp. |
|  | OTU393 | -1.206 | 0.7778 | Basidiomycota | *Rhodotorula vanillica* |
|  | OTU396 | -1.302 | 0.7500 | Ascomycota | *Acremonium nepalense* |
|  | OTU397 | -0.998 | 0.7600 | Zygomycota | *Mortierella camargensis* |
|  | OTU406 | 0.000 | 0.7500 | Basidiomycota | Unclassified |
|  | OTU412 | -1.608 | 0.6875 | Ascomycota | *Fusarium* sp. |
|  | OTU413 | -1.415 | 0.7500 | Basidiomycota | *Cystofilobasidium infirmominiatum* |
|  | OTU522 | -0.998 | 0.6875 | Ascomycota | Unclassified |
|  | OTU669 | 0.000 | 0.7778 | Ascomycota | *Westerdykella dispersa* |
|  | OTU671 | 0.426 | 0.6296 | Basidiomycota | *Leucosporidiales* sp. |
|  | OTU710 | -1.092 | 0.7500 | Ascomycota | *Gibberella zeae* |
|  | OTU715 | 0.000 | 0.7778 | Ascomycota | *Stagonospora perfecta* |
|  | OTU717 | -1.023 | 0.7778 | Ascomycota | Unclassified |
| LS | OTU1587 | -1.690 | 0.7500 | Ascomycota | *Clonostachys rosea f. catenulata* |
|  | OTU298 | 0.539 | 0.6296 | Ascomycota | *Westerdykella dispersa* |
|  | OTU2981 | -0.671 | 0.6533 | Ascomycota | *Preussia flanaganii* |
|  | OTU2994 | -1.558 | 0.7551 | Ascomycota | *Phoma calidophila* |
|  | OTU301 | -0.866 | 0.7500 | Basidiomycota | Unclassified |
|  | OTU3337 | -1.155 | 0.7500 | Ascomycota | *Thelebolales* sp. |
|  | OTU3473 | -0.671 | 0.6533 | Zygomycota | *Mortierella camargensis* |
|  | OTU3545 | -0.257 | 0.7500 | Ascomycota | *Thelebolales* sp. |
|  | OTU361 | -0.227 | 0.6508 | Zygomycota | Unclassified |
|  | OTU375 | -1.598 | 0.7778 | Ascomycota | *Fusarium tricinctum* |
|  | OTU381 | -2.169 | 0.6875 | Zygomycota | Unclassified |
|  | OTU383 | -1.598 | 0.7778 | Ascomycota | *Cordyceps bassiana* |
|  | OTU409 | -0.671 | 0.6533 | Ascomycota | *Pyrenochaetopsis leptospora* |
|  | OTU434 | -2.001 | 0.7500 | Ascomycota | *Scutellinia torrentis* |
|  | OTU476 | 1.155 | 0.7500 | Ascomycota | *Neurospora terricola* |
|  | OTU520 | -1.558 | 0.6944 | Basidiomycota | Unclassified |
|  | OTU697 | -1.441 | 0.6451 | Ascomycota | Unclassified |
|  | OTU708 | 0.642 | 0.6875 | Incertaesedis | *Sclerotium hydrophilum* |
|  | OTU726 | -0.257 | 0.7500 | Zygomycota | *Mortierella humilis* |
|  | OTU927 | -2.001 | 0.7778 | Ascomycota | Unclassified |

**Table S5:** The potential pathogens were detected in guts of hooded cranes.

| Pathogenic species | Symptom | Infected target |
| --- | --- | --- |
| *Sarocladium strictum* | Meningitis, pneumonia | Human, horse ^[1, 2]^ |
| *Talaromyces marneffei* | Talaromycosis, pneumonia, lymphadenitis | Human, mice, dog ^[3-5]^ |
| *Gibberella fujikuroi* | Fusariosis, hepatitis | Human, bird, poultry ^[6, 7]^ |
| *Fusarium tricinctum* | Dermatitis, stomatitis | Human, chicken, bird ^[8, 9]^ |
| *Westerdykella dispersa* | Sarcoidosis | Human, dog ^[10]^ |
| *Rhodotorula vanillica* | Fungemia | Human ^[11]^ |
| *Penicillium oxalicum* | Bronchitis | Human ^[12]^ |

**Reference:**

1. Cui Y, Meng JL, Zhang J, Wang L, Yan HH, Xia H, Cao JR, Wu LY. Case Report: A Case of *Sarocladium strictum* Meningoencephalitis in an Immunocompetent Patient After Invasive Operation. Frontiers in Medicine. 2021; 8. doi: 10.3389/fmed.2021.762763
2. Pusterla N, Holmberg T A, Lorenzo-Figueras M, Wong A, Wilson WD. *Acremonium strictum* pulmonary infection in a horse. Veterinary Clinical Pathology. 2005; 34: 413-416. doi: 10.1111/j.1939-165X.2005.tb00072.x
3. Pruksaphon K, Nosanchuk JD, Ratanabanangkoon K, Youngchim S. *Talaromyces marneffei* Infection: Virulence, Intracellular Lifestyle and Host Defense Mechanisms. Journal of Fungi. 2022; 8: 200. doi: 10.3390/jof8020200
4. Liu YH, Huang XW, Yi XW, He Y, Mylonakis E, Xi LY. Detection of *Talaromyces marneffei* from fresh tissue of an inhalational murine pulmonary model using nested PCR. PloS one. 2016; 11: e0149634. doi: 10.1371/journal.pone.0149634
5. Seyedmousavi S, Guillot J, Tolooe A, Verweij PE, de Hoog GS. Neglected fungal zoonoses: hidden threats to man and animals. Clinical Microbiology and Infection. 2015; 21: 416-425. doi: 10.1016/j.cmi.2015.02.031
6. Tortorano AM, Prigitano A, Esposto MC, Arsenijevic VA, Kolarovic J, Ivanovic D, Paripovic L, Klingspor L, Nordoy I, Hamal P, Akdagli SA, Ossi C, et al. European Confederation of Medical Mycology (ECMM) epidemiological survey on invasive infections due to *Fusarium* species in Europe. European Journal of Clinical Microbiology & Infectious Diseases. 2014; 33: 1623-1630. doi: 10.1007/s10096-014-2111-1
7. Bailly JD, Benard G, Jouglar JY, Durand S, Guerre P. Toxicity of *Fusarium moniliforme* culture material containing known levels of fumonisin B1 in ducks. Toxicology. 2001; 163: 11-22. doi: 10.1016/S0300-483X(01)00368-7
8. O'Donnell K, Sutton DA, Wiederhold N, Robert VARG, Crous PW, Geiser DM. Veterinary fusarioses within the United States. Journal of Clinical Microbiology. 2016; 54: 2813-2819. doi: 10.1128/JCM.01607-16
9. Ademoyero AA, Hamilton PB. Mouth lesions in broiler chickens caused by scirpenol mycotoxins. Poultry science. 1991; 70: 2082-2089. doi: 10.3382/ps.0702082
10. Sue PK, Gurda GT, Lee R, Watkins T, Green R, Memon W, Milstone AM, Zelazny AM, Fahle GA, Pham TA, Gibas CF, Sutton DA, et al. First report of *Westerdykella dispersa* as a cause of an angioinvasive fungal infection in a neutropenic host. Journal of Clinical Microbiology. 2014; 52: 4407-4411. doi: 10.1128/JCM.02012-14
11. Wirth F, Goldani LZ. Epidemiology of Rhodotorula: An Emerging Pathogen. Interdisciplinary Perspectives on Infectious Diseases. 2012; 2012: 465717. doi: 0.1155/2012/465717
12. Chowdhary A, Kathuria S, Agarwal K, Sachdeva N, Singh PK, Jain S, Meis JF. Voriconazole-resistant *Penicillium oxalicum*: an emerging pathogen in immunocompromised hosts. Open Forum Infectious Diseases. 2014; 1. doi: 10.1093/ofid/ofu029


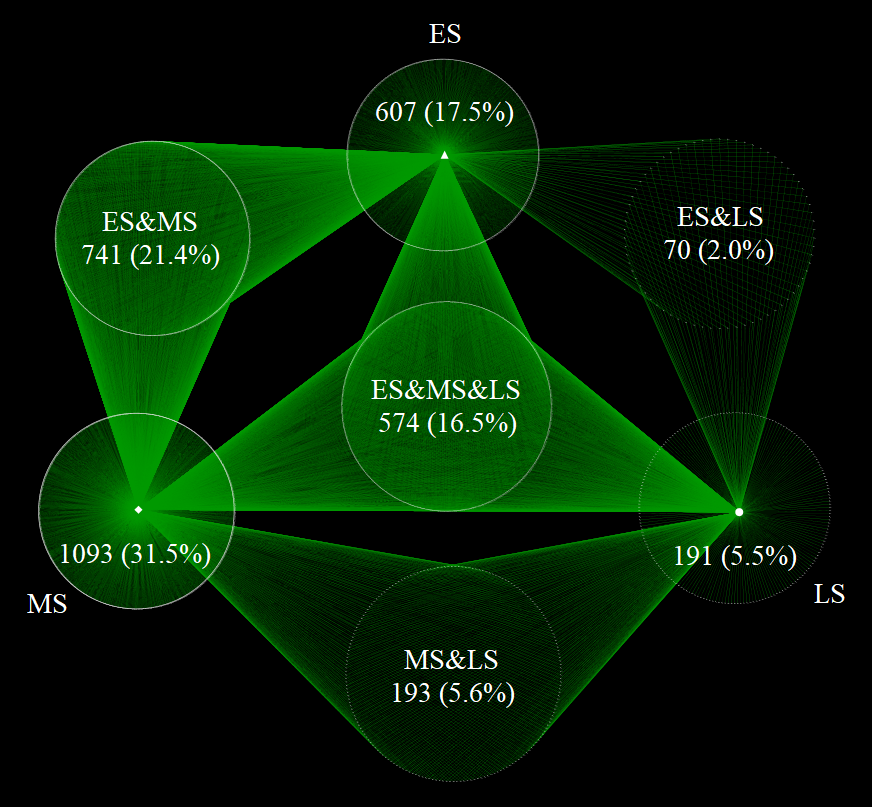


**Figure S1:** The Venn diagram showing the unique and shared gut fungal OTUs in guts of hooded cranes among three wintering stages. ES: early stage; MS: middle stage; LS: late stage. OTU: operational taxonomic unit.


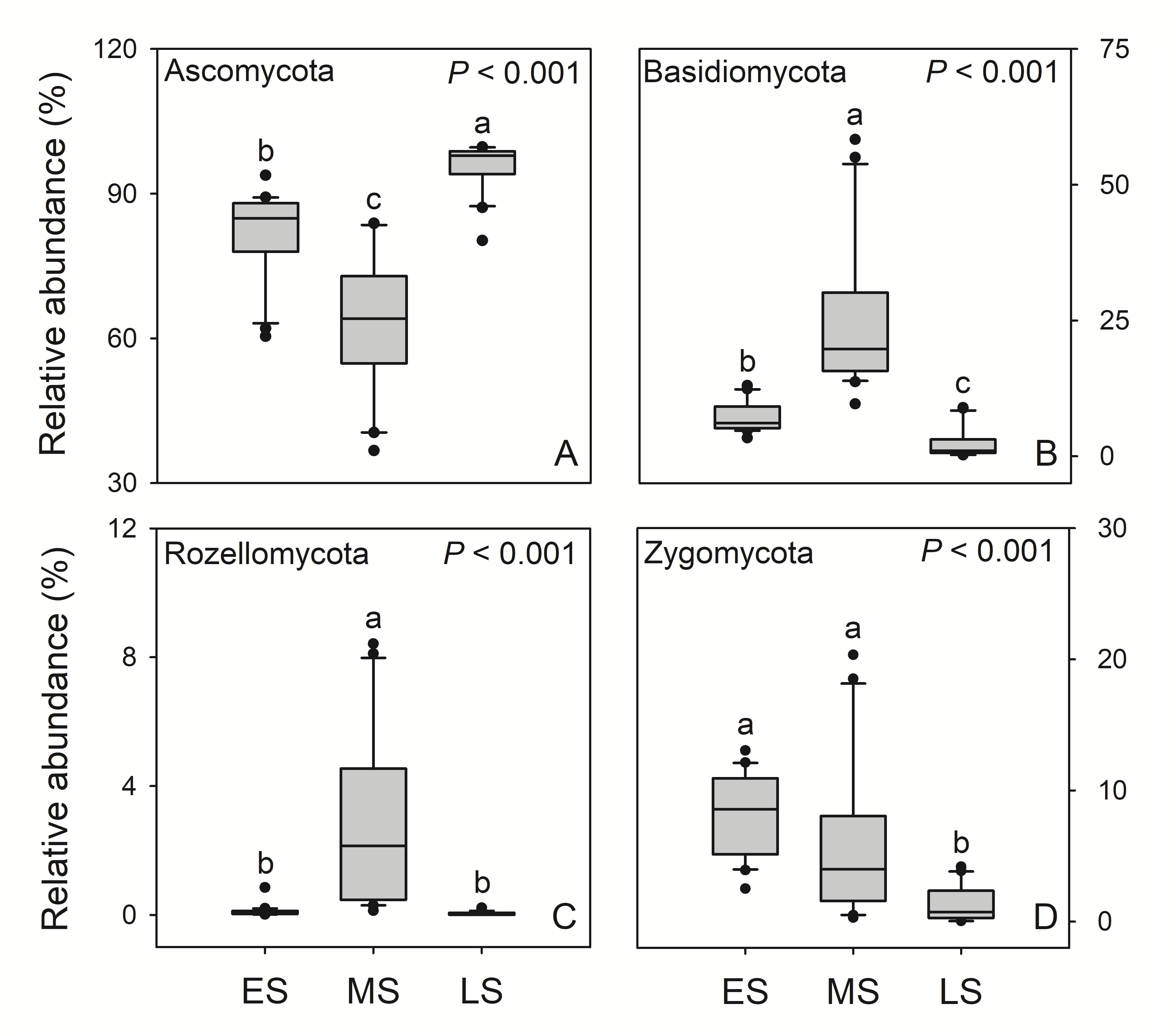


**Figure S2:** Relative abundance of the dominant fungal phyla. The bottom and top of the box denote the first and third quartiles; the band inside the box denotes median. ES: early stage; MS: middle stage; LS: late stage.


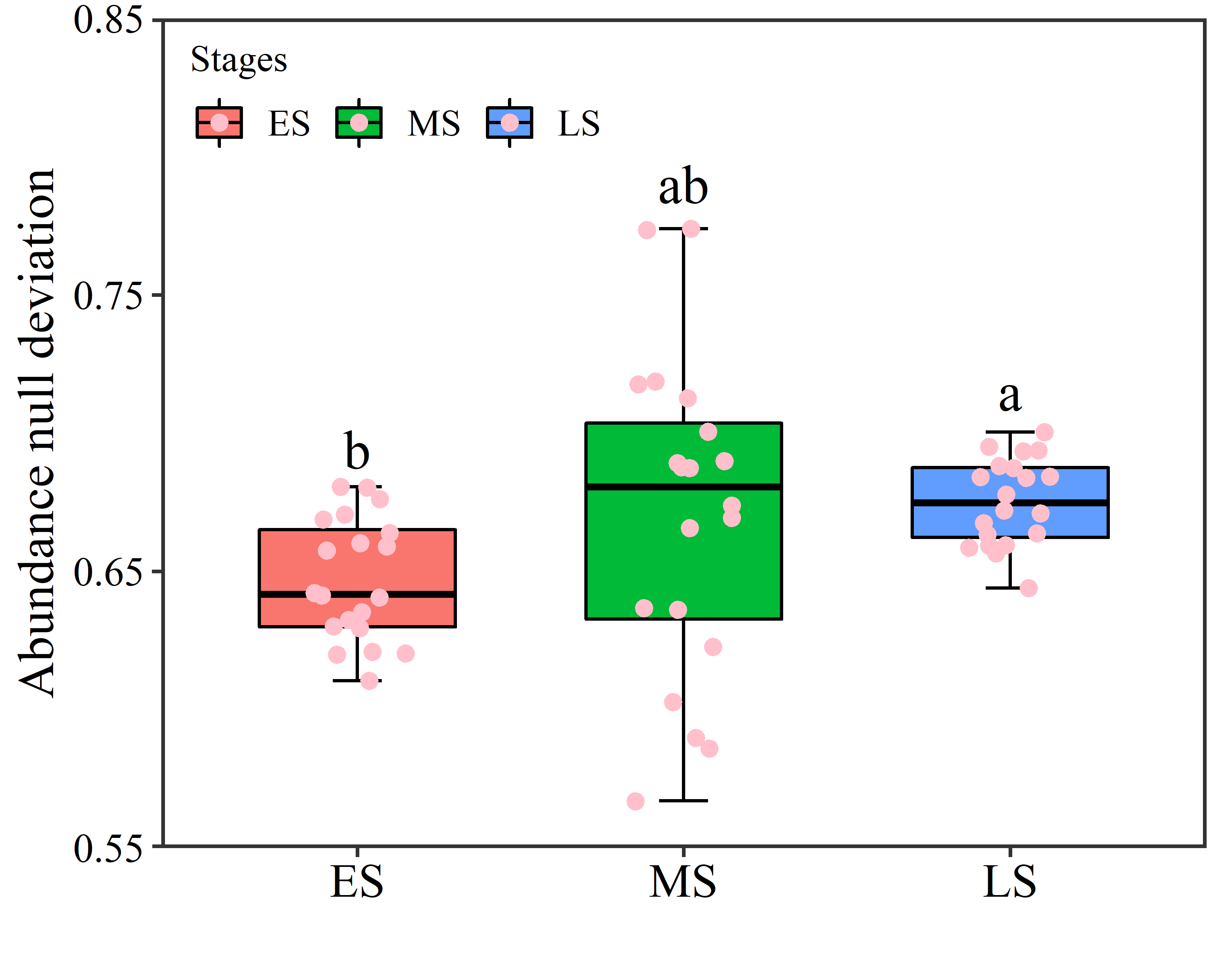


**Figure S3:** The abundance-based β-null model was used to distinguish the relative importance of deterministic and stochastic processes by the value of null deviation. A β-diversity deviation value closer to zero indicates higher stochasticity, whereas a β-diversity deviation value closer to 1 indicates higher deterministicity. Bars with different lowercase letters indicate significant differences. ES: early stage; MS: middle stage; LS: late stage.


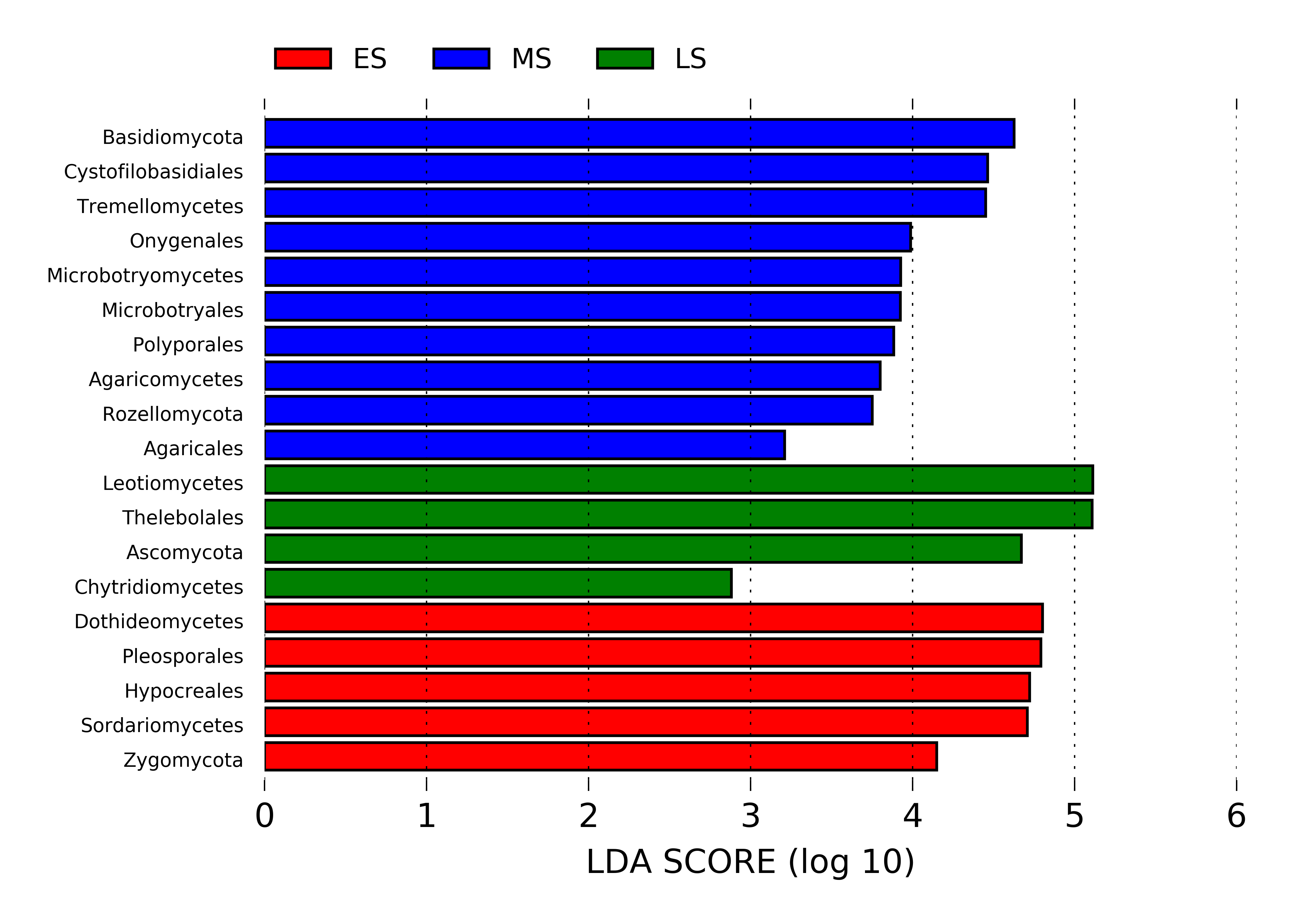


**Figure S4:** Identified phylotype biomarkers ranked by effect size with the alpha value was < 0.05 in different wintering stages. The phylotype biomarkers were identified as significantly abundant when samples from early, middle and late stages were compared. ES: early stage; MS: middle stage; LS: late stage.


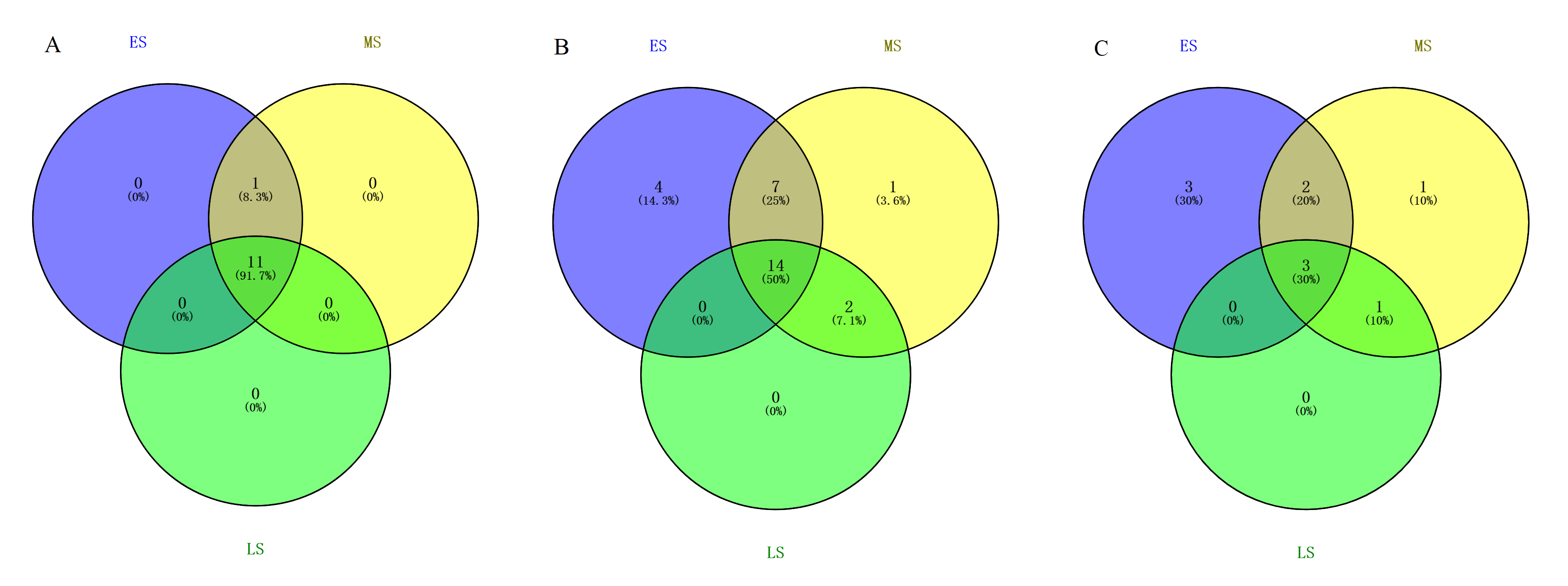


**Figure S5:** Venn diagram showing the unique and shared plant saprotrophic OTUs (A), animal pathogenic OTUs (B) and endosymbiotic OTUs (C) in guts of the hooded cranes in different stages. ES: early stage; MS: middle stage; LS: late stage.


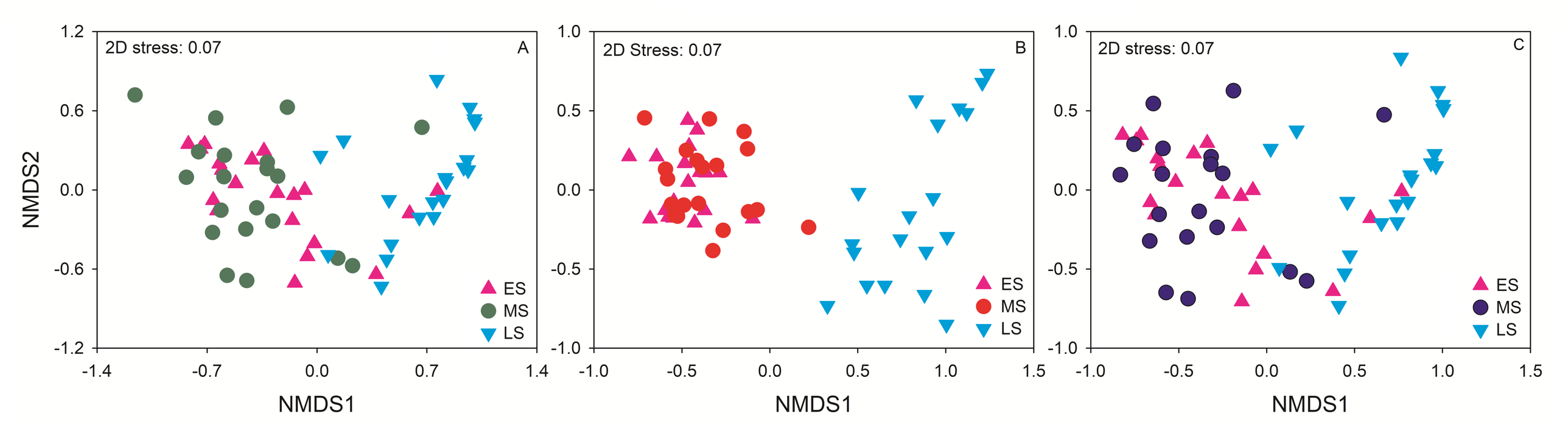


**Figure S6:** The intestinal plant saprotrophic community composition (A), animal pathogenic community composition (B) and endosymbiotic community composition (C) among the three stages. ES: early stage; MS: middle stage; LS: late stage.


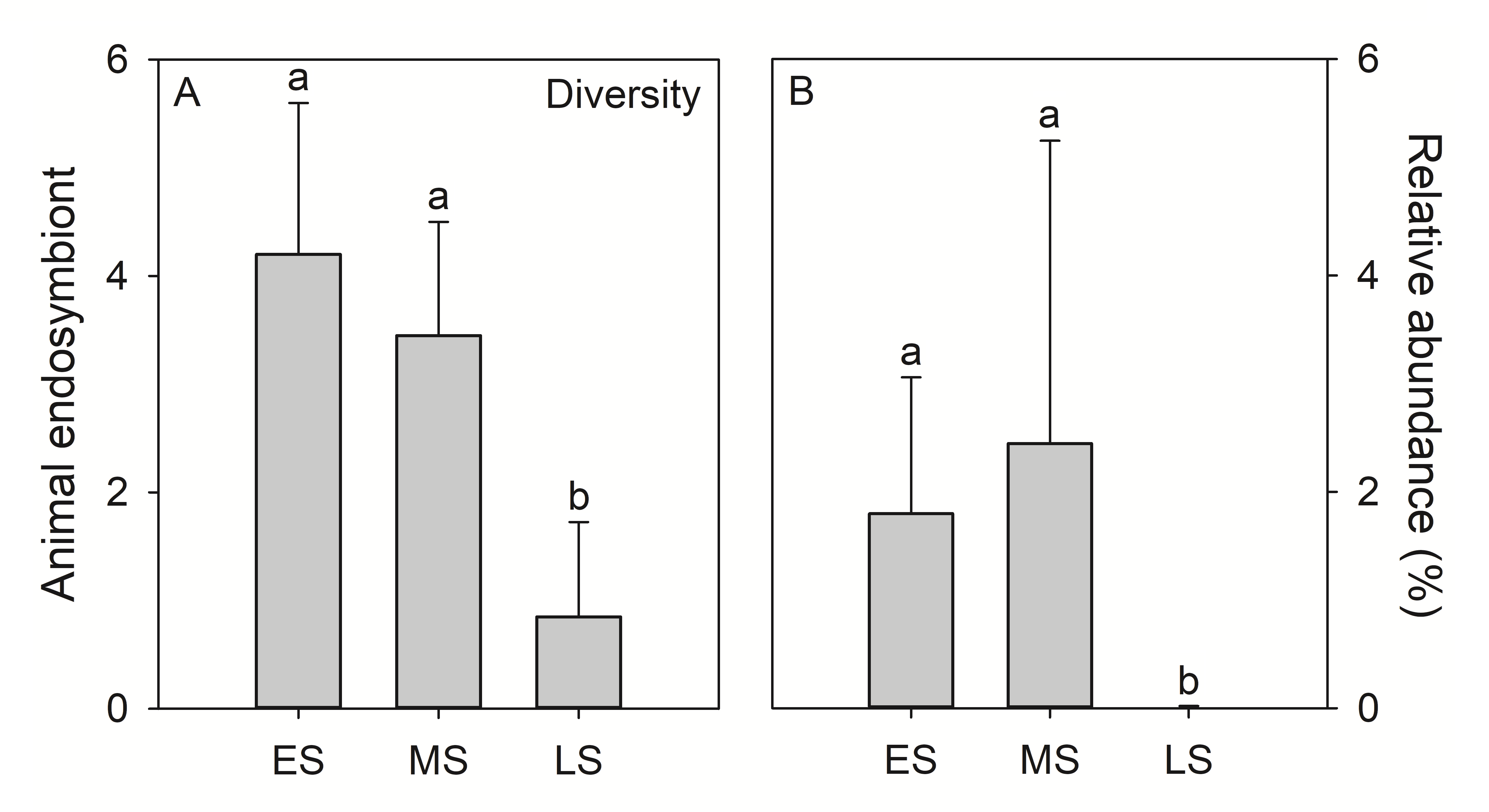


**Figure S7:** The animal endosymbiotic diversity (i.e., endosymbiotic OTU; A) and the relative abundance of animal endosymbiont (B). Letters represent significant differences from the Kruskal-Wallis test (*P* < 0.05). ES: early stage; MS: middle stage; LS: late stage. OTU: operational taxonomic units.


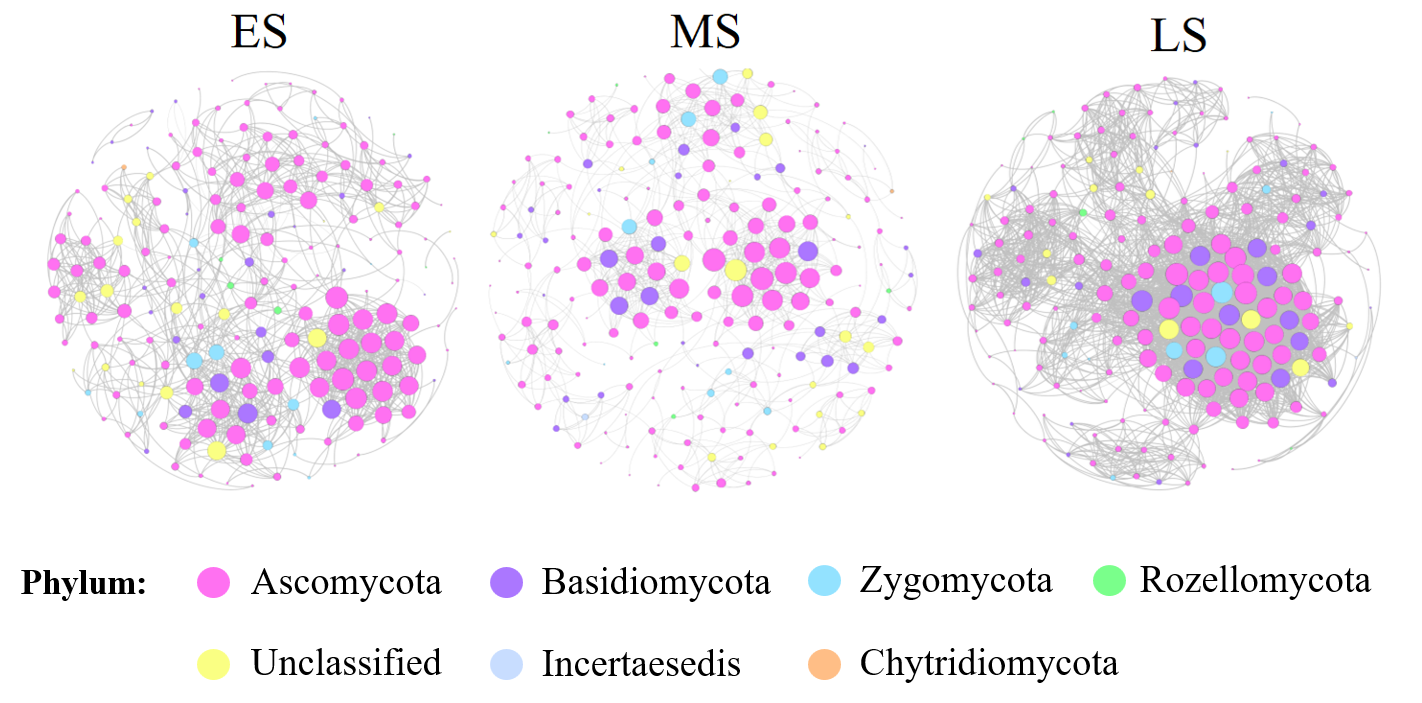


**Figure S8:** The co-occurrence network structure of gut fungal community for hooded cranes with OTU’s relative abundance > 0.01% at phylum level among three stages. ES: early stage; MS: middle stage; LS: late stage.


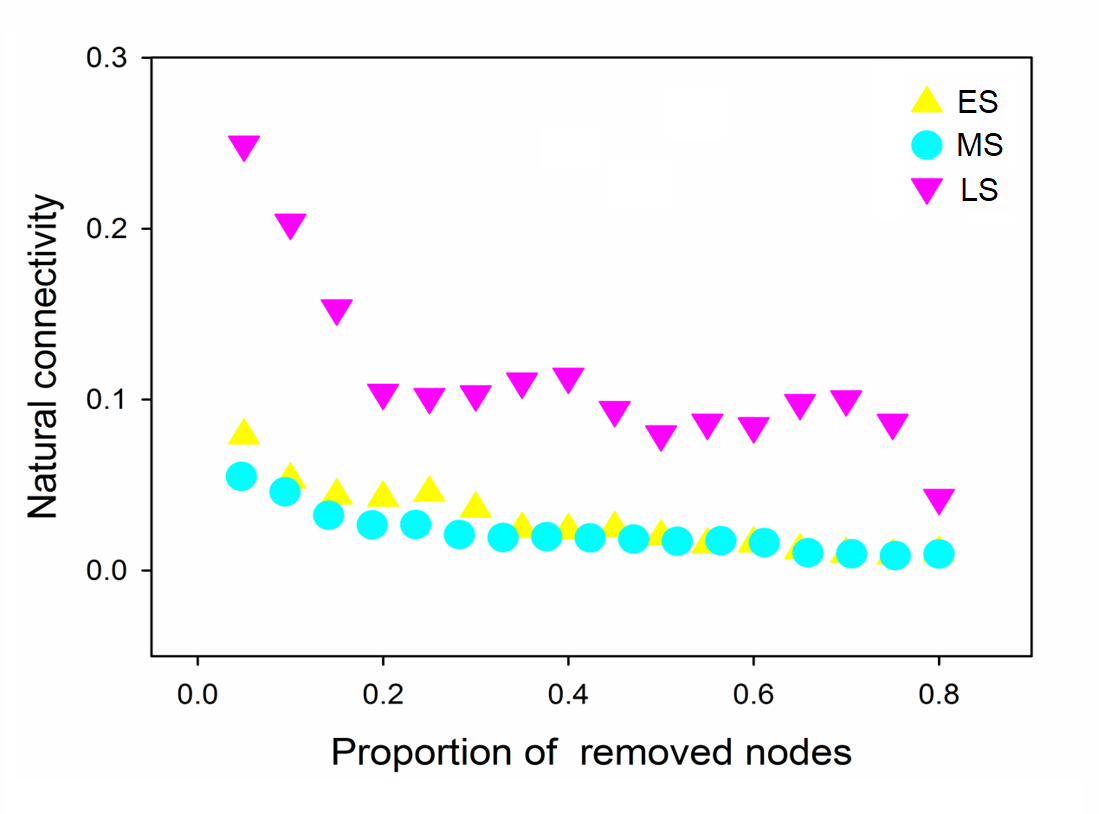


**Figure S9:** The stability of co-occurrence network. ES: early stage; MS: middle stage; LS: late stage.


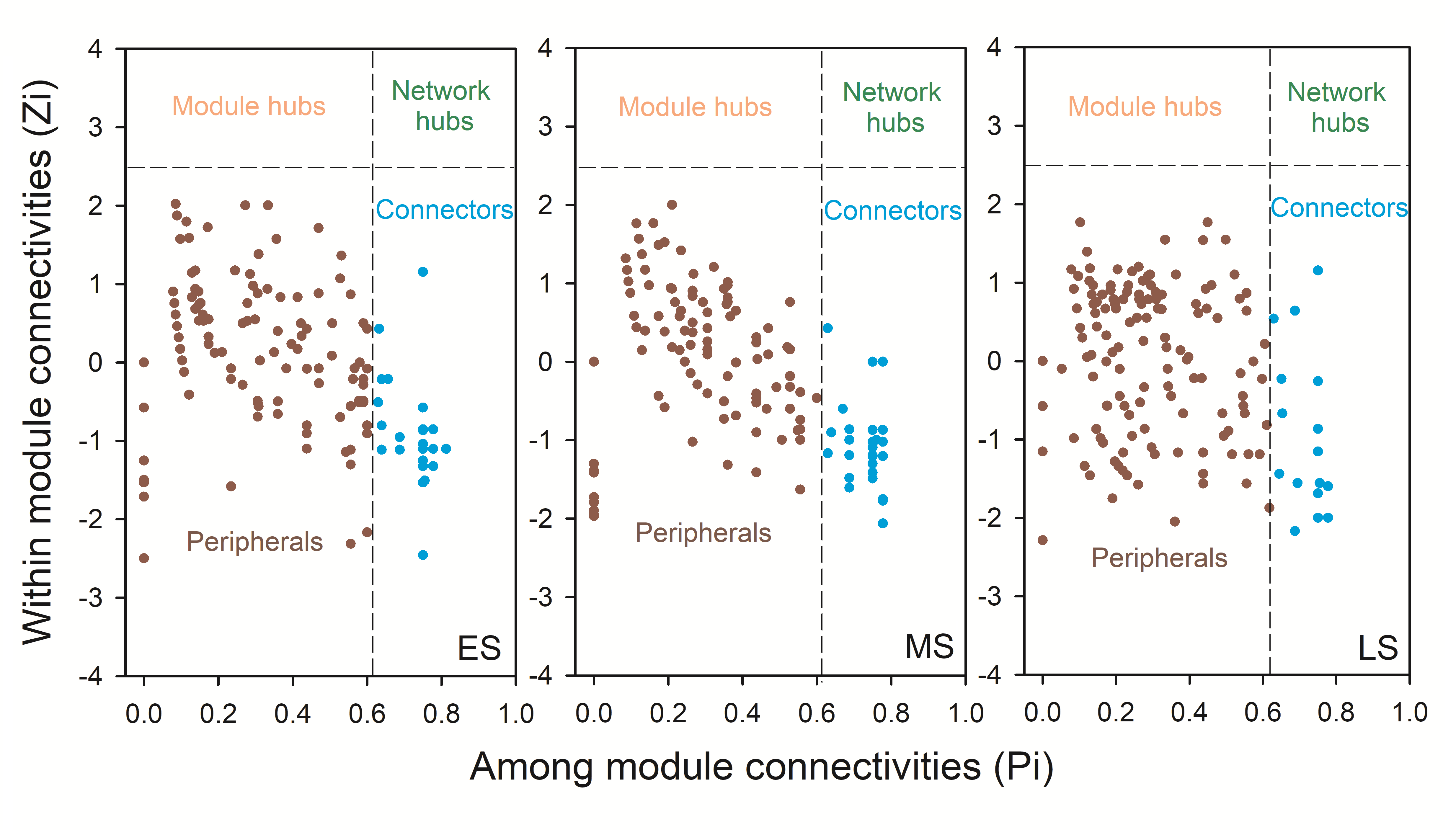


**Figure S10:** The network roles of analyzing module feature at OTU level with relative abundance > 0.01% among three stages. The Zi and Pi thresholds for OTU classification were 2.5 and 0.62, respectively. ES: early stage; MS: middle stage; LS: late stage.
